# Loss of Active Neurogenesis in the Adult Shark Retina

**DOI:** 10.3389/fcell.2021.628721

**Published:** 2021-02-11

**Authors:** Ismael Hernández-Núñez, Diego Robledo, Hélène Mayeur, Sylvie Mazan, Laura Sánchez, Fátima Adrio, Antón Barreiro-Iglesias, Eva Candal

**Affiliations:** ^1^Departamento de Bioloxía Funcional, Facultade de Bioloxía, CIBUS, Universidade de Santiago de Compostela, Santiago de Compostela, Spain; ^2^The Roslin Institute and Royal (Dick) School of Veterinary Studies, University of Edinburgh, Edinburgh, United Kingdom; ^3^CNRS, Sorbonne Universités, UPMC Univ Paris 06, UMR7232, Observatoire Océanologique, Banyuls-sur-mer, France; ^4^Departamento de Zooloxía, Xenética e Antropoloxía Física, Facultade de Veterinaria, Universidade de Santiago de Compostela, Lugo, Spain

**Keywords:** retina, neurogenesis, cell proliferation, RNA-seq, mitosis, progenitor cells, cartilaginous fish

## Abstract

Neurogenesis is the process by which progenitor cells generate new neurons. As development progresses neurogenesis becomes restricted to discrete neurogenic niches, where it persists during postnatal life. The retina of teleost fishes is thought to proliferate and produce new cells throughout life. Whether this capacity may be an ancestral characteristic of gnathostome vertebrates is completely unknown. Cartilaginous fishes occupy a key phylogenetic position to infer ancestral states fixed prior to the gnathostome radiation. Previous work from our group revealed that the juvenile retina of the catshark *Scyliorhinus canicula*, a cartilaginous fish, shows active proliferation and neurogenesis. Here, we compared the morphology and proliferative status of the retina in catshark juveniles and adults. Histological and immunohistochemical analyses revealed an important reduction in the size of the peripheral retina (where progenitor cells are mainly located), a decrease in the thickness of the inner nuclear layer (INL), an increase in the thickness of the inner plexiform layer and a decrease in the cell density in the INL and in the ganglion cell layer in adults. Contrary to what has been reported in teleost fish, mitotic activity in the catshark retina was virtually absent after sexual maturation. Based on these results, we carried out RNA-Sequencing (RNA-Seq) analyses comparing the retinal transcriptome of juveniles and adults, which revealed a statistically significant decrease in the expression of many genes involved in cell proliferation and neurogenesis in adult catsharks. Our RNA-Seq data provides an excellent resource to identify new signaling pathways controlling neurogenesis in the vertebrate retina.

## Introduction

Neurogenesis is the process by which multipotent neural stem cells (NSCs) generate new neurons. In the mammalian central nervous system (CNS), multipotent NSCs are known as apical progenitors and include neuroepithelial cells (NECs) that have limited capacity for self-renewal and divide symmetrically to generate more NECs, and apical radial glial cells (RGCs) that divide symmetrically or asymmetrically to generate neurons and glial cells, sometimes through secondary progenitors known as apical intermediate progenitor cells (IPCs; Namba and Huttner, 2017). Basal progenitors are all secondary progenitors that include basal RGCs and basal IPCs that divide once or several times to amplify a cell lineage (Namba and Huttner, 2017). As CNS develops, neurogenesis becomes progressively reduced to specific areas called neurogenic niches (Grandel and Brand, 2013; Ganz and Brand, 2016), where it persists during postnatal life. Despite this decline in the neurogenic potential, new functional neurons have been found in the adult brain of all vertebrate groups studied, which are generated from adult NSCs. Adult neurogenesis recapitulates to some extent the neurogenic process occurring in early development, but certain regulatory mechanisms are unique to the process in adults. Moreover, adult neurogenesis via these neurogenic niches is highly dependent on species and CNS regions, i.e., different types of progenitors with different neurogenic potentials have been described depending on the neurogenic niche (Docampo-Seara et al., 2020 and references therein; Jurkowski et al., 2020).

Teleost fishes display the most prominent and widespread adult neurogenesis throughout the CNS compared with any other vertebrate studied so far (Ganz and Brand, 2016 and references therein). From anamniotes to amniotes, constitutive neurogenesis in adults become restricted to fewer and more anterior neurogenic niches (Grandel and Brand, 2013). The fact that the retina of teleost fishes shows active proliferation and neurogenic activity throughout life (goldfish: Johns, 1977; rainbow trout: Julian et al., 1998; zebrafish: Marcus et al., 1999) together with its cytoarchitectonic simplicity and amenability to manipulation provided the basis for the use of the teleost retina as a model for the study of adult neurogenesis in vertebrates. Two main niches of constitutive neurogenesis, which can also contribute to regeneration of the damaged retina, have been described in the retina of adult teleosts: the ciliary marginal zone (CMZ), which is a continuously growing circumferential ring of cells located in the peripheral part of the retina (Johns, 1977; Stenkamp et al., 2001), and Müller glial cells located in the central (mature) retina (Fausett and Goldman, 2006; Raymond et al., 2006; Bernardos et al., 2007; Lenkowski and Raymond, 2014).

While the localization of adult neurogenic niches shows an outstanding conservation among teleost species (Ganz and Brand, 2016), the localization of neurogenic niches within the retina of different vertebrates could not be so well-conserved. Actually, the retina of adult lampreys (a jawless vertebrate) shows no proliferative activity (Villar-Cheda et al., 2008). Constitutive proliferation within the CMZ has been also described in adult frogs, turtles and birds, and in young marsupials (Straznicky and Gaze, 1971; Fischer and Reh, 2000; Kubota et al., 2002; Todd et al., 2016 and references therein), though it has been reported that CMZ cells diminished during vertebrate evolution (Kubota et al., 2002). Müller glia are proliferative under physiological conditions only in fishes (reviewed in Sánchez-Farías, 2016). In addition, some vertebrate groups show neurogenesis in the adult retina from constitutive niches other than the CMZ and Müller glial cells [such as a pseudostratified region at the retinociliary junction between the retina and the ciliary body in some species of lizards and snakes (Eymann et al., 2019) or the pigment epithelium of the ciliary body of rodents (Tropepe et al., 2000)], and from other potential sources of adult neurogenesis that have been unveiled by regeneration studies (for a revision on sources of retinal regeneration in vertebrates see Amato et al., 2004; Moshiri et al., 2004; Fischer et al., 2013; Chiba, 2014; Fernández-Nogales et al., 2019; García-García et al., 2020). Besides, in fishes, data on adult neurogenesis in the retina come only from studies in modern teleost models (e.g., zebrafish or goldfish) but there is no data on adult neurogenesis in more basal teleosts, other ray-finned fishes (e.g., chondrosteans) or in chondrichthyans (cartilaginous fishes, the oldest extant jawed vertebrates). Therefore, a better understanding of adult neurogenesis in a representative sampling of vertebrates, including groups which diverged early from the osteichthyan lineage, will be essential to gain knowledge on the evolution of adult neurogenesis in the vertebrate retina.

As the sister group of osteichthyans, cartilaginous fishes occupy a key phylogenetic position to infer ancestral states fixed prior to the gnathostome radiation or to identify lineage-specific diversifications in the major osteichthyan taxa. Our research group has thoroughly studied the development of the retina of an elasmobranch fish, the catshark *Scyliorhinus canicula*. The embryonic retina of *S. canicula* shows a high proliferative activity that decreases throughout development, although proliferation is still observed in juveniles (Ferreiro-Galve et al., 2010). An interesting feature of the retina of *S. canicula* is that it presents a region adjacent to the CMZ, called the transition zone (TZ), which also contains progenitor cells (Ferreiro-Galve et al., 2010, 2012; Sánchez-Farías and Candal, 2015, 2016). Different types of progenitor cells have been identified in the CMZ and TZ both in the developing and in the postnatal retina of *S. canicula* based on the expression of progenitor cell markers like proliferating cell nuclear antigen (PCNA), phosphohistone H3 (pH3), doublecortin (DCX), or glial fibrillary acidic protein (GFAP), though both DCX and GFAP also label postmitotic cells (Ferreiro-Galve et al., 2010, 2012; Sánchez-Farías and Candal, 2015, 2016). In adults, DCX was shown to be expressed in postmitotic neurons (Sánchez-Farías and Candal, 2015) while GFAP was found in the ciliary epithelium, the CMZ, and in mature Müller glial cell processes in the central retina (Sánchez-Farías and Candal, 2016). However, whether active proliferation and neurogenesis is maintained in the adult retina has not been addressed so far.

Here, we aimed to gain knowledge about the evolution of adult neurogenesis in the vertebrate retina by exploring this process in cartilaginous fishes with the catshark *Scyliorhinus canicula* as reference. We characterized changes in the morphology and neurogenic ability of the retina of *S. canicula* from postnatal development to adulthood using classical histological staining and by analyzing the expression of proliferation markers. Additionally, we carried out an Illumina RNA-sequencing (RNA-Seq) study comparing the retinal transcriptomes of juveniles and adults to detect changes in the expression of genes involved in cell proliferation and neurogenesis, and to identify new genes and signaling pathways responsible for the neurogenic process in the retina of vertebrates.

## Materials and Methods

### Animals

Juvenile (*n* = 17; 10–13 cm long) and adult specimens [*n* = 15; 45–50 cm long, which correspond to sexually mature animals (Kousteni and Megalofonou, 2020)] of *S. canicula* were kindly provided by the aquarium *Acuario do Grove* (O Grove, Spain) and kept in seawater tanks under standard conditions of temperature (15–16°C), pH (7.5–8.5), and salinity (35 g/L). All experimental procedures were performed following the guidelines established by the European Union and the Spanish government for animal experimentation and were approved by the Bioethics Committee of the University of Santiago de Compostela.

### Tissue Preparation for Histology

Animals were deeply anesthetized with 0.5 % tricaine methanesulfonate (MS-222, Sigma, St. Louis, MO) in seawater and then perfused intracardially with elasmobranch Ringer's solution (see Ferreiro-Galve et al., 2008) followed by perfusion with 4% paraformaldehyde (PFA) in 0.1 M elasmobranch phosphate buffer (pH 7.4) containing 1.75% urea. The eyes were removed and postfixed in 4% PFA for 2 days at 4°C. After rinsing in phosphate buffer saline (PBS), the eyes were cryoprotected with 30% sucrose in PBS, embedded in Neg-50^TM^ (Thermo Scientific, Kalamazoo, MI), and frozen with liquid nitrogen-cooled isopentane. Transverse sections (18 μm thick) were obtained on a cryostat and mounted on Superfrost Plus slides (Menzel-Glasser, Madison, WI).

### Haematoxylin-Eosin Staining

Some retinal sections were stained with haematoxylin-eosin following standard protocols. Briefly, cryostat sections were dried at room temperature (RT), rinsed in 0.05 M Tris-buffered (pH 7.4) saline (TBS) for 10 min and stained with haematoxylin solution for 10 min. Sections were subsequently rinsed in tap water until removal of the excess of haematoxylin, in distilled water for 10 min and then stained with eosin for 2 min. Finally, the sections were dehydrated and mounted in DPX mounting medium (Scharlau, Sentmenat, Spain).

### Immunofluorescence

Sections were first pre-treated with 0.01 M citrate buffer pH 6.0 for 30 min at 90°C for heat-induced epitope retrieval, allowed to cool for 20 min at RT and rinsed in TBS for 5 min. Then, sections were incubated overnight at RT with different combinations of primary antibodies [(1) rabbit polyclonal anti-GFAP (1:500; DakoCytomation, Denmark; Z0334) and mouse monoclonal anti-glutamine synthase (GS; 1:500; Millipore, Billerica, MA; Mab302); (2) mouse monoclonal anti-PCNA (1:500; Sigma; P8825) and rabbit polyclonal anti-pH3 (1:300; Millipore; 06-570)], or rabbit polyclonal anti-DCX (1:300; Cell Signaling Technology, Danvers, MA; 4604). Sections were rinsed three times in TBS for 10 min each, and incubated with the fluorescent dye-labeled secondary antibodies Cy3-conjugated goat anti-rabbit (1:200; Sigma; A10520) and FITC-conjugated goat anti-mouse (1:200; Sigma; F2761) for 1 h at RT. All antibody dilutions were made in TBS containing 15% normal goat serum (Millipore), 0.2% Triton X-100 (Sigma), and 2% BSA (Sigma). Sections were then rinsed three times in TBS for 10 min each and in distilled water for 30 min, allowed to dry for 30 min at 37°C, and mounted in MOWIOL 4-88 Reagent (Calbiochem, Darmstadt, Germany).

### Specificity of Antibodies

The anti-GFAP and the anti-GS antibodies have been previously used in the retina of the juvenile catshark as markers of early and late radial glial cells, respectively (Sánchez-Farías and Candal, 2015, 2016). Their specificity has been tested by western blot in brain protein extracts of adult catsharks (Docampo-Seara et al., 2019). PCNA is present in proliferating cells and although its expression is stronger during the S phase, it persists along the entire cell cycle excepting the mitotic period (Zerjatke et al., 2017). The anti-PCNA antibody has been previously used to label progenitor cells in the brain and retina of *S. canicula* (i.e., Quintana-Urzainqui et al., 2014; Sánchez-Farías and Candal, 2015). The anti-pH3 antibody has been also widely used in the brain and retina of *S. canicula* as a marker of mitotic cells (Ferreiro-Galve et al., 2010; Quintana-Urzainqui et al., 2014; Docampo-Seara et al., 2019). The anti-DCX antibody has been previously used in the retina of the developing and adult catshark retina as a marker for neuronal lineage (Sánchez-Farías and Candal, 2015, 2016). Its specificity has been tested by western blot in brain protein extracts of adult catsharks (Pose-Méndez et al., 2014).

### Riboprobe Synthesis and *in situ* Hybridization

*In situ* hybridization (ISH) experiments were carried out to study the expression of *S. canicula Sox2* (*ScSox2*) transcripts using the probe described in Lagadec et al. (2018). Sense and antisense digoxigenin-UTP-labeled *ScSox2* riboprobes were synthesized by *in vitro* transcription, after PCR amplification, and using the T7 or SP6 RNA polymerase (NZYTech, Lisbon, Portugal). ISH was performed on cryostat sections (18 μm) of juvenile and adult retinas as previously described (Coolen et al., 2007). Briefly, sections were permeabilized with proteinase K, hybridized with sense or antisense probes overnight at 65°C and incubated with the alkaline phosphatase-coupled sheep anti-digoxigenin antibody (1:2000, Roche Applied Science, Manheim, Germany) overnight at 4°C. The color reaction was performed using BM-Purple (Roche). Finally, sections were dehydrated and mounted in DPX. Control sense probes did not produce any detectable signal.

### Image Acquisition

Images of fluorescent labeled sections were taken with a Leica TCS-SP2 confocal microscope with a combination of blue and green excitation lasers. Confocal optical sections were taken at steps of 1 μm along the z-axis. Collapsed images were obtained with the LITE software (Leica). Brightfield images were obtained with an Olympus BX51 microscope equipped with an Olympus DP71 camera. Contrast and brightness were minimally adjusted using Adobe Photoshop CS4 (Adobe, San Jose, CA).

### Cell Quantifications and Statistical Analyses

Retinal area, thickness of the inner nuclear layer (INL) and inner plexiform layer (IPL), and cell density of the INL and ganglion cell layer (GCL) were quantified in haematoxylin-eosin-stained retinal sections (*n* = 3 retinas from three different juveniles; *n* = 3 retinas from three different adults) using Image J (Schneider et al., 2012). For the quantification of retinal area, 10 sections were quantified in each retina. For the quantification of layer thickness and cell density, five sections were quantified in each retina. Area is given as mm^2^, thickness as mm and cell density as the number of nuclei in a 50 × 50 μm square. Then, we calculated the mean area, layer thickness, and cell density for each retina.

We quantified the number of mitotic [pH3 positive (pH3+) cells] in the peripheral retina (CMZ and TZ) and proliferative cells (PCNA+ cells) in the whole central retina of juveniles (*n* = 6 retinas from six different individuals) and adults (*n* = 5 retinas from five different individuals). The number of pH3+ cells and PCNA+ cells were manually counted under the microscope in one out of each four consecutive retinal sections (18 μm). The limit between the peripheral and the central retina was established based on morphological differences like the characteristic layered structure of the central retina. Then, we calculated the mean number of cells per section for each retina.

Statistical analyses were performed with Prism 8 (GraphPad software, La Jolla, CA). Normality of the data was determined with the Shapiro-Wilk test. To determine significant differences (*p* < 0.05) between juveniles and adults, a Mann-Whitney test was used for non-normally distributed data (only the pH3+ data) and a Student's (unpaired) *t*-test was used for normally distributed data.

### RNA Isolation and Sequencing

The retinas of juveniles (*n* = 6 retinas from five animals) and adults (*n* = 5 retinas from four animals) were dissected out and put in RNAlater (Ambion Inc., Austin, TX, USA). RNA extraction was performed using the RNeasy mini kit (Qiagen, Hilden, Germany) with DNase treatment following the manufacturer's instructions. Isolated RNAs were eluted in nuclease free water. RNA quality and quantity were evaluated in a Bioanalyzer (Bonsai Technologies, Madrid, Spain) and in a NanoDrop® ND-1000 spectrophotometer (NanoDrop® Technologies Inc., Wilmington, DE, USA). Thereafter, the Illumina Truseq mRNA stranded RNA-Seq Library Prep Kit protocol was followed. Libraries were checked for quality and quantified using the Bioanalyzer 2100 (Agilent, Santa Clara, CA, USA) (all samples had RIN values higher than seven), before being sequenced on one S1 lane of the Illumina NovaSeq instrument using 150 base paired-end sequencing at Edinburgh Genomics (UK). Raw reads have been deposited in NCBI's Sequence Read Archive (SRA) under BioProject accession number PRJNA668789.

The quality of the sequencing output was assessed using FastQC v.0.11.5 (http://www.bioinformatics.babraham.ac.uk/projects/fastqc/). Quality filtering and removal of residual adaptor sequences was conducted on read pairs using Fastp v.0.20.0 (Chen et al., 2018). Illumina specific adaptors were clipped from the reads and leading and trailing bases with a Phred score <20 were removed; only reads where both pairs were longer than 36 bp post-filtering were retained. Filtered reads were mapped to a catshark transcriptomic reference obtained by a clustering approach (Mayeur and Mazan, to be published elsewhere) and transcript abundance was quantified using Kallisto v0.46.1 (Bray et al., 2016).

Differential expression (DE) analyses were performed using R v.3.6.2 (https://www.r-project.org/). Gene count data were used to estimate differential gene expression using the Bioconductor package DESeq2 v.3.4 (Love et al., 2014). Briefly, size factors were calculated for each sample using the “median of ratios” method and count data was normalized to account for differences in library depth. Next, gene-wise dispersion estimates were fitted to the mean intensity using a parametric model and reduced toward the expected dispersion values. Finally, a negative binomial model was fitted for each gene and the significance of the coefficients was assessed using the Wald test. The Benjamini-Hochberg false discovery rate (FDR) multiple test correction was applied, and genes with FDR < 0.01, normalized mean read counts > 50 and absolute log2 fold change values (FC) > 1 were considered differentially expressed. Gene Ontology (GO) and Kyoto Encyclopedia of Genes and Genomes (KEGG) enrichment were performed using the DAVID bioinformatics resource Functional Annotation tool (Huang et al., 2009a), using the shark transcriptome as background.

## Results

### Comparison of Juvenile and Adult Retinal Morphology

**Figure 1:**
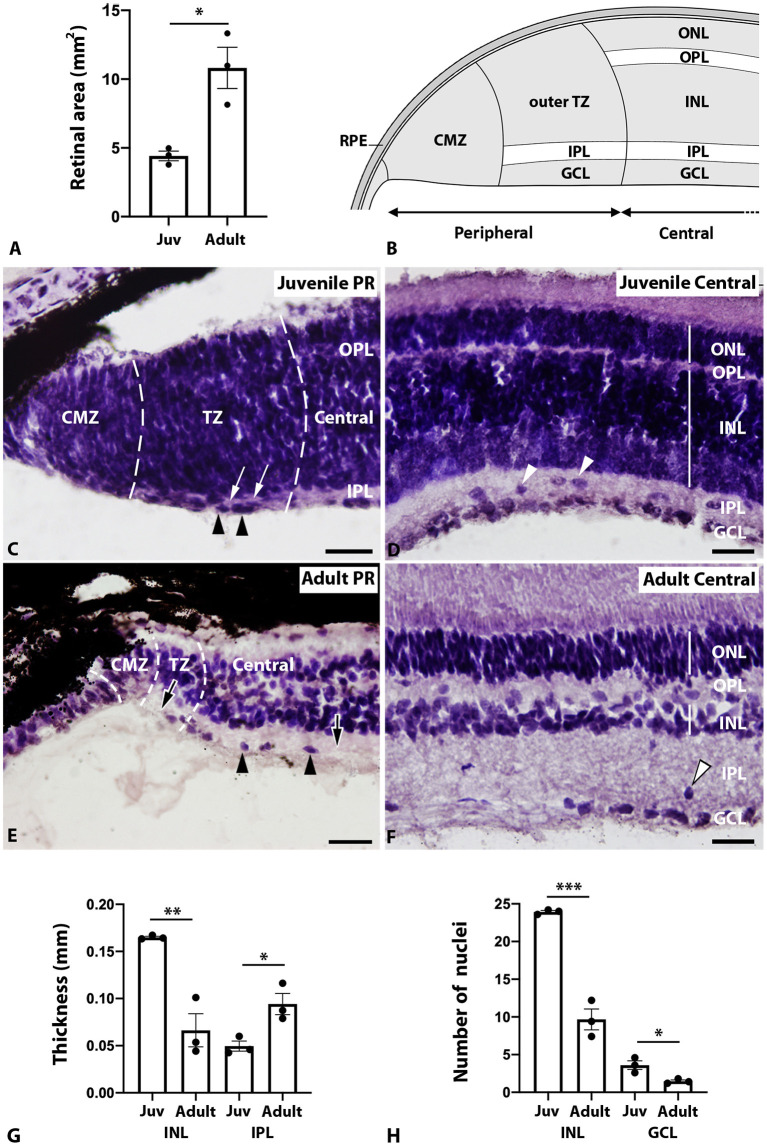
Morphological changes between the juvenile and adult retinas. **(A)** Graph showing a significant change (*p* = 0.0142; *) in retinal area between juveniles (Juv; 4.42 ± 0.35 mm^2^) and adults (10.82 ± 1.5 mm^2^). **(B)** Schematic drawing showing the different regions and layers in the juvenile peripheral and central retina. **(C)** Juvenile peripheral retina showing: an unlayered CMZ; a TZ, where the IPL (white arrows) separates the unlayered outer region from the GCL (black arrowheads); and the central retina, where the two plexiform layers (OPL and IPL) can be observed. **(D)** Juvenile central retina showing its characteristic layers (ONL, OPL, INL, IPL, GCL) and displaced cells in the IPL (white arrowheads). **(E)** The adult peripheral retina showed a reduction in size with respect to the juvenile retina. Note the increase in the thickness of the IPL in the TZ and in the central retina (arrows). Black arrowheads point to cells in the GCL. **(F)** The adult central retina showed an evident decrease in the thickness of the INL, an increase in the thickness of the IPL and a decrease in cell density in the INL and GCL. Displaced cells were observed in the IPL (arrowhead). **(G)** Graph showing significant changes in the thickness of the INL (*p* = 0.005; **; juveniles: 0.16 ± 0.001 mm; adult: 0.06 ± 0.01 mm) and IPL (*p* = 0.0231; *; juveniles: 0.05 ± 0.005 mm; adults: 0.09 ± 0.01 mm). **(H)** Graph showing significant changes in cell density of the INL (*p* = 0.0005; ***; juveniles: 23.93 ± 0.18 nuclei/50 × 50 μm square; adults: 9.67 ± 1.39 nuclei/50 × 50 μm square) and GCL (*p* = 0.0241; *; juveniles: 3.6 ± 0.58 nuclei/50 × 50 μm square; adult: 1.47 ± 0.18 nuclei/50 × 50 μm square). Each dot in the graphs represents one retina and all retinas are from different animals. Scale bars: 100 μm **(C)**; 50 μm **(D–F)**. CMZ, ciliary marginal zone; GCL, ganglion cell layer; INL, inner nuclear layer; IPL, inner plexiform layer; Juv, juvenile; ONL, outer nuclear layer; OPL, outer plexiform layer; PR, peripheral retina; RPE, retinal pigment epithelium; TZ, transition zone.

The adult retina increased in size respect to the juvenile retina as revealed by area quantification in retinal sections (Figure 1A; *p* = 0.0142). As previously reported (Ferreiro-Galve et al., 2010), the peripheral retina of juveniles showed a non-layered CMZ with a neuroepithelial organization and a TZ where the IPL separates an unlayered, outer, region from the GCL (Figures 1B,C). As in lampreys and jawed vertebrates, the catshark central retina of juveniles was composed of three nuclear layers [the outer nuclear layer (ONL), the INL, and the GCL] and two plexiform layers [the outer plexiform layer (OPL) and the IPL] (Figures 1B,D). In adults, the peripheral retina showed the same zones (CMZ and TZ) observed in juveniles but with an important reduction in size and extension when compared to the juvenile retina and also relative to the total size of the adult retina (Figure 1E). The adult central retina also showed the typical layered organization (Figure 1F), but we observed a significant decrease in the thickness of the INL (Figures 1D,F,G; *p* = 0.005), an increase in the thickness of the IPL (Figures 1D,F,G; *p* = 0.0231) and a significant decrease in cell density in the INL and GCL (Figures 1D,F,H; INL: *p* = 0.0005; GCL: *p* = 0.0241).

### Defining the Peripheral Retina

To better define the border of the peripheral retina, where the main retinal neurogenic niche in *S. canicula* is located, we studied the expression pattern of two glial cell markers (GFAP and GS) and the neural stem and progenitor cell marker Sox2.

**Figure 2:**
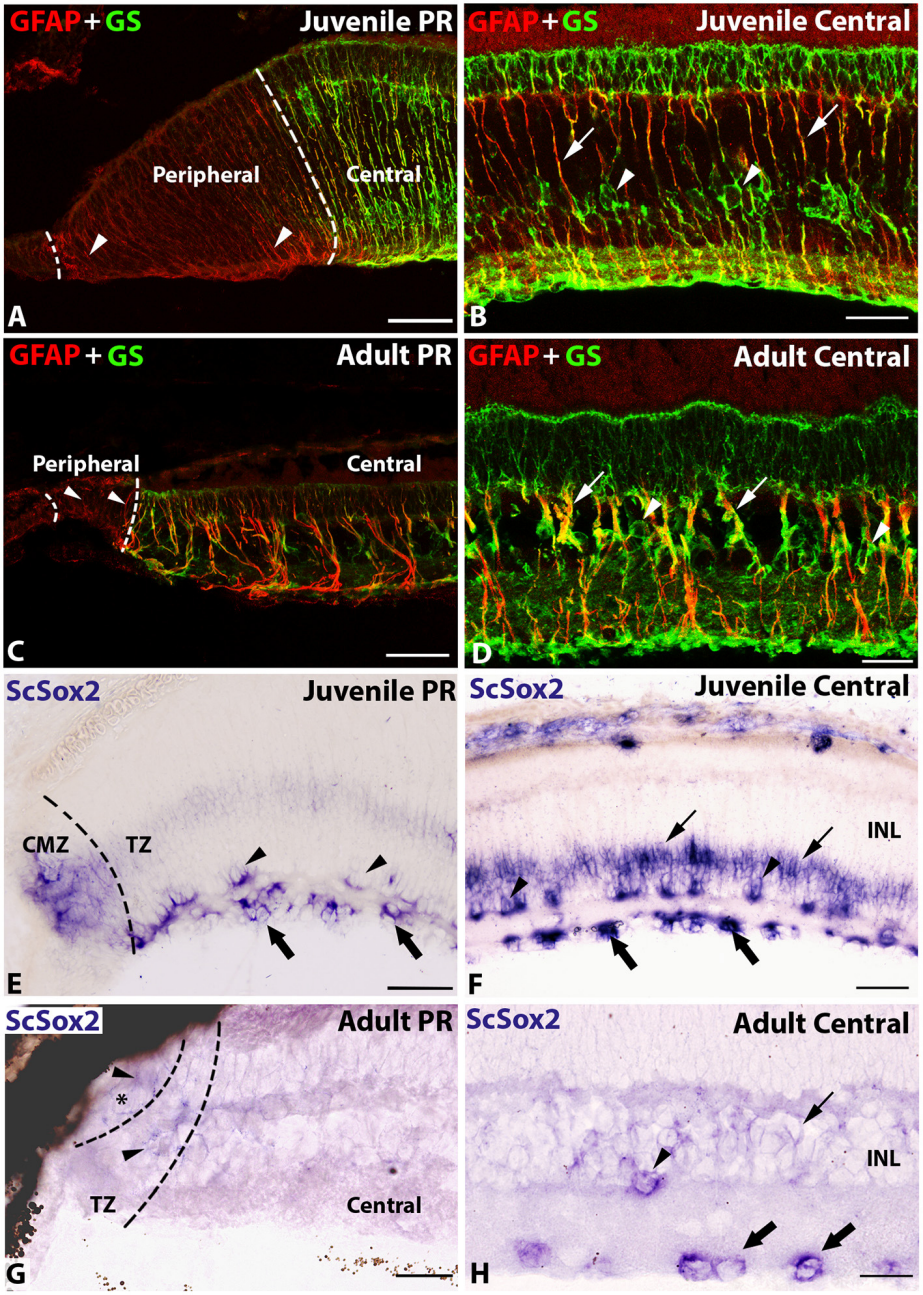
Double labeling with GFAP and GS **(A–D)** and *in situ* hybridization of *ScSox2* **(E–H)** in transverse sections of the retina of juvenile and adult specimens of *S. canicula*. **(A)** In the juvenile retina, GFAP was expressed in processes of progenitor cells in the peripheral retina (arrowheads) while GS expression was not observed in this region. **(B)** In the juvenile central retina, co-expression of GFAP and GS was observed in processes (arrows) and GS expression was observed in cell bodies (arrowheads) of Müller glia. **(C)** In the adult peripheral retina, GFAP+ putative progenitor cells were found in the retinal periphery (arrowheads). **(D)** In the adult central retina, as in juveniles, GFAP+ and GS+ Müller glia processes (arrows) and GS+ Müller glia cell bodies (arrowheads) were observed. **(E)** *ScSox2* expression was observed in the CMZ and inner region of the TZ of the juvenile peripheral retina. Note the presence of amacrine (arrowheads) and ganglion (thick arrows) cells. **(F)** *ScSox2* expression was observed in amacrine (arrowheads), ganglion (thick arrows), and Müller glia (arrows) cells of the juvenile central retina. **(G)** *ScSox2* expression was much weaker (arrowheads) in the adult peripheral retina than in juveniles. The asterisk indicates the CMZ. **(H)** In the adult central retina, *ScSox2* was expressed in amacrine (arrowhead), ganglion (thick arrows), and Müller glial cells (arrow), though expression levels decreased with respect to juveniles. Scale bars: 100 μm **(A,E)**; 50 μm **(B–D, F–H)**. CMZ, ciliary marginal zone; INL, inner nuclear layer; PR, peripheral retina; TZ, transition zone.

In the juvenile retina, GFAP expression was observed in processes of progenitor cells in the peripheral retina (Figure 2A; Sánchez-Farías and Candal, 2016) and in mature Müller glial cells in the central retina (Figures 2A,B; Sánchez-Farías and Candal, 2016). However, GS expression was restricted to processes and cell bodies of mature Müller glial cells in the central retina (Figures 2A,B; Sánchez-Farías and Candal, 2016), which facilitates delimiting the boundary between the peripheral and central retinas (Figure 2A).

In adults (Figures 2C,D), the expression patterns of GFAP and GS were similar to those observed in juveniles (Figures 2A,B), and also allowed for a clear identification of peripheral and central regions of the retina (Figures 2C,D). The size of the peripheral retina containing GFAP+ cells in adults was highly reduced with respect to juveniles and relative to the size of the entire retina (Figure 2C).

Sox2 is known to be expressed in progenitor cells that keep their stem cells properties in adults (DeOliveira-Mello et al., 2019) and in postmitotic amacrine, ganglion, and Müller glia cells (Surzenko et al., 2013; DeOliveira-Mello et al., 2019). *ScSox2 in situ* labeling allowed us to clearly establish the boundary between the CMZ and TZ within the peripheral retina in juvenile specimens (Figure 2E). An intense expression of *ScSox2* was observed in the CMZ and the inner region of the TZ (Figure 2E), and in amacrine, ganglion, and Müller glia cells in the central retina of juveniles (Figure 2F). The expression of *ScSox2* in the adult retina (Figures 2G,H) clearly decreased with respect to that observed in juveniles (Figures 2E,F). In the adult peripheral retina, a very weak labeling was observed in cells of the CMZ and TZ (Figure 2G). In the adult central retina, *ScSox2* labeling was observed in amacrine, ganglion, and Müller glia cells (Figure 2H).

### Proliferation, Mitotic Activity, and Neurogenesis

**Figure 3:**
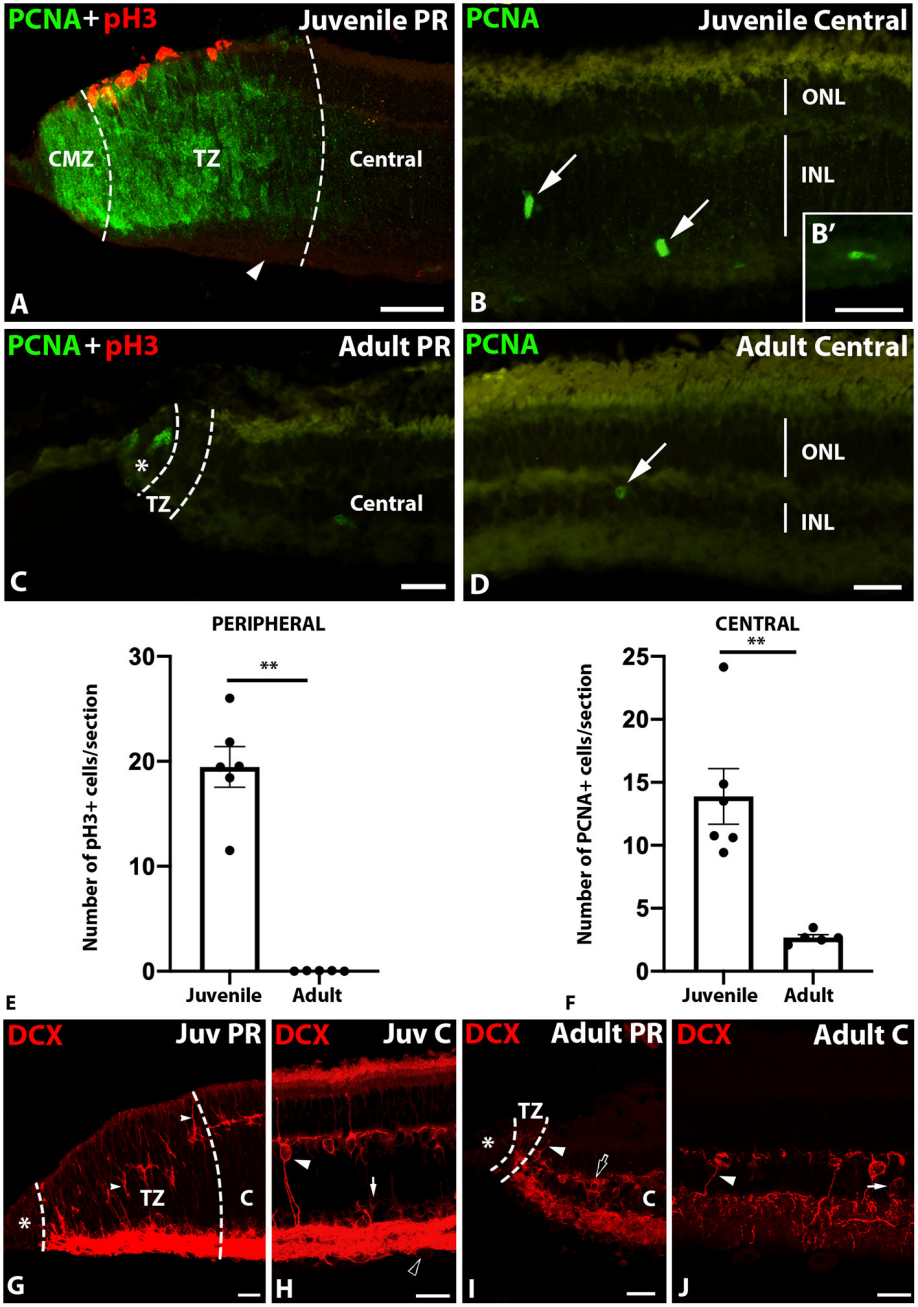
Double labeling with PCNA and pH3 **(A,C)** and PCNA **(B,D)** in transverse sections of the retina of juvenile and adult specimens of *S. canicula*, graphical representations of the average number of mitotic **(E)** and proliferative cells **(F)** per section in both stages and DCX immunofluorescence in transverse sections of the retina of juvenile **(G,H)** and adult **(I,J)** specimens of *S. canicula*. **(A)** Abundant PCNA+ cells were observed in the CMZ and the TZ of juveniles. Note the absence of PCNA+ cells in the GCL in the TZ (arrowhead). Mitotic cells (pH3+) were only found in the ventricular (apical) region. (**B,B'**) In the central retina of juveniles, PCNA was found in scattered cells in the INL (arrows) and in displaced cells within the IPL **(B')**. **(C)** In adults, only a few PCNA+ cells were observed in the apical region of the CMZ (asterisk), while almost no pH3+ cells were found in this region. **(D)** Occasional PCNA+ cells were found in the adult central retina (arrow). **(E)** Graph showing the average number of mitotic cells (pH3+) per section in the peripheral retina of juveniles and adults revealing statistically significant differences in the number of mitotic cells (*p*-value = 0.0043; **; juveniles: 19.47 ± 1.94 cells per section; adults: 0.03 ± 0.01 cells per section). **(F)** Graph showing the average number of proliferating cells (PCNA+) per section in the central retina of juveniles and adults revealing statistically significant differences in the number of proliferating cells (*p*-value = 0.0014; **; juveniles: 13.89 ± 2.21 cells per section; adults: 2.67 ± 0.22 cells per section). Each dot in the graphs represents one retina and all retinas are from different animals. **(G)** In the juvenile peripheral retina, DCX expression was absent in the pre-neurogenic CMZ (asterisk) while a strong DCX immunoreactivity was observed in cell bodies and radial processes of the TZ (arrowheads). **(H)** In the juvenile central retina DCX was found in subsets of mature horizontal (not shown), bipolar (white arrowhead), amacrine (white arrow), and ganglion cells (black arrowhead). **(I)** In the adult peripheral retina, the neurogenic TZ containing DCX+ radial cell processes was highly reduced in size. The asterisk indicates the CMZ. The white arrowhead points to a bipolar cell. The black arrow points to a horizontal cell. **(J)** In the adult central retina, as in juveniles, DCX was expressed in mature cells, including bipolar cells (white arrowhead) and amacrine cells (white arrow). Scale bars: 100 μm **(A)**; 50 μm **(B–D)**; 25 μm **(G–J)**. C, central retina; CMZ, ciliary marginal zone; INL, inner nuclear layer; Juv, juvenile; ONL, outer nuclear layer; PR, peripheral retina; TZ, transition zone.

It is well-known that the proliferative potential of the retina decreases during development in vertebrates; nevertheless, cell proliferation is still observed in adult teleost fishes (see section Introduction). Here, we compared the proliferative status and mitotic activity of juvenile and adult catshark retinas using PCNA and pH3 double immunolabeling. As previously reported (Ferreiro-Galve et al., 2010), in the peripheral retina of juveniles abundant PCNA+ cells were found throughout the CMZ and the outer non-layered part of the TZ, but not in the prospective GCL (Figure 3A). pH3 positive (pH3+) cells were restricted to the ventricular (apical) region of the peripheral retina (Figure 3A). In the central retina of juveniles, scattered PCNA+ cells were found in the INL (Figure 3B), bordering the horizontal cell layer (HCL; not shown) and in the GCL/IPL (Figure 3B'). No pH3+ cells were found in the central retina of juveniles (not shown).

In the peripheral retina of adults only occasional PCNA+ cells were observed, most of them being restricted to the CMZ (Figure 3C). Interestingly, almost no pH3+ cells were observed in this region (not shown). Only occasional PCNA+ cells were observed in the central retina of adults (Figure 3D). As in juveniles, no pH3+ cells were found in this region (not shown).

Quantifications of mitotic (pH3+) and proliferating (PCNA+) cells revealed significant differences in the proliferative capacity of juvenile and adult retinas. There was a statistically significant decrease in the number of pH3+ cells (*p* = 0.0043) in the peripheral retina of adults (Figure 3E). We also observed a statistically significant decrease in the number of PCNA+ cells in the central retina of adults (*p* = 0.0014, Figure 3F). The pH3 results reveal a dramatic loss of mitotic activity in the peripheral adult retinas.

To further asses the neurogenic activity of the peripheral retina we performed DCX immunolabeling. We previously showed that DCX immunoreactivity in the developing retina of elasmobranch fishes was absent from pre-neurogenic NECs located in the most peripheral part of the CMZ. In the remaining peripheral retina, DCX expression increases in RGCs coinciding with the onset of neurogenesis and in neuroblasts before they become definitely positioned (Sánchez-Farías and Candal, 2015, 2016). DCX was also found in subsets of mature horizontal, bipolar, amacrine, and ganglion cells in the central retina, where it persists in adults (Sánchez-Farías and Candal, 2015). As previously reported, in juveniles, DCX was not observed in the pre-neurogenic CMZ, while a strong DCX immunoreactivity was observed in cell bodies and radial processes of the neurogenic TZ (Figure 3G) and in mature cells in the central retina (Figure 3H). In adults, the neurogenic TZ was highly reduced in size (Figure 3I). As in juveniles, DCX was found in mature cells in the central retina (Figure 3J).

### RNA-Sequencing

Our results revealed important differences between the juvenile and the adult retina mainly regarding the huge decrease of proliferative activity in the peripheral and central retina, with an almost complete lack of mitotic activity in the retina of adult specimens.

**Figure 4:**
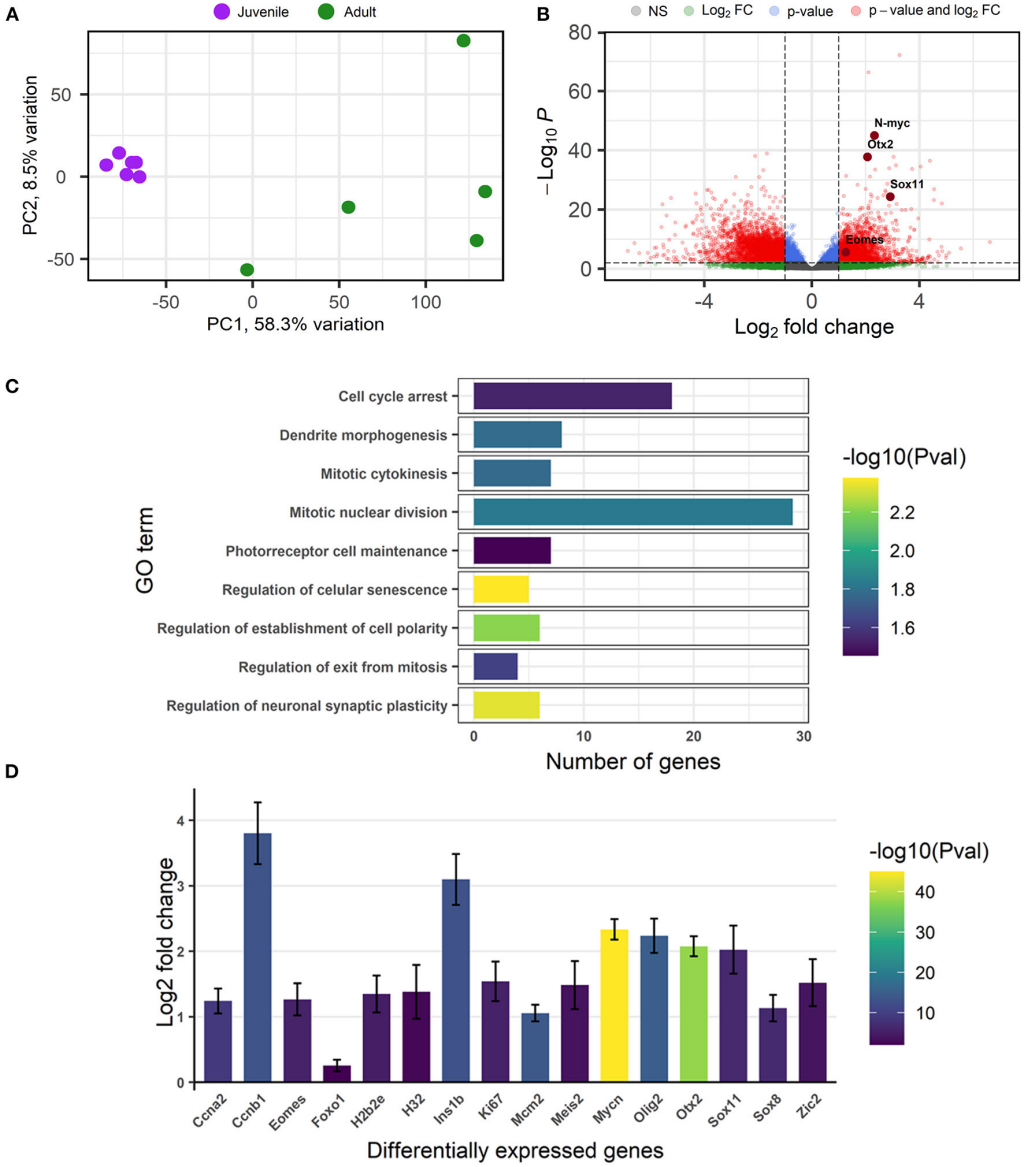
RNA-Seq analysis of the retinal transcriptome **(A–D)**. **(A)** Principal component analysis showing the juvenile (purple dots) and adult (green dots) samples clustered according to their gene expression. Each dot represents one retina. **(B)** Volcano plot displaying the results of the RNA-seq analysis (statistical significance vs. magnitude of change) and highlighting significant genes. Each dot represents one gene. A positive fold change indicates a decreased expression in the adult retina. Four of the genes that showed decreased expression in the adult retina (*Eomes, Otx2, N-myc*, and *Sox11*) are highlighted. **(C)** Graph showing some of the significantly enriched pathways (GO terms) indicating the number of genes significantly changing their expression in each pathway. **(D)** Graph showing some of the genes with a significantly decreased expression in adults. The color code in **(C,D)** indicates the *p*-value (Pval). FC, fold change; NS, not significant; PC, principal component.

In order to identify the genes and signaling pathways responsible for the high proliferative activity of the juvenile retina compared to the quiescence observed in adults, we performed an Illumina RNA-Seq analysis comparing the retinal transcriptomes of juveniles and adults. The juvenile and adult retinal transcriptomes showed marked differences and clustered clearly apart in the principal component analysis (Figure 4A). The first principal component, separating the juvenile and adult samples, explained a very large proportion of the variance (58.3%). Juvenile retinas formed a tight cluster, whereas adult retinas showed more variation. Since the exact age of adult animals is not known, this variation in the principal component analysis could be attributed to higher differences in the age of the adult individuals. In any case, the sexually mature adult samples are clearly separated from sexually immature juvenile samples. A total of 6,359 differentially expressed genes were detected (Figure 4B; Supplementary Material 1). Of these, 4,203 genes showed decreased expression and 2,156 showed increased expression in the adult retina (Supplementary Material 1). GO term and KEGG pathway enrichment analyses showed that many of the significantly enriched pathways are related to cell division and cell cycle arrest (GO terms: “mitotic nuclear division,” “mitotic cytokinesis,” “regulation of exit from mitosis,” and “cell cycle arrest”), neuronal plasticity (GO terms: “regulation of neuronal synaptic plasticity” and “dendrite morphogenesis”) or other developmental pathways (GO terms: “regulation of establishment of cell polarity,” “photoreceptor cell maintenance,” and “regulation of cellular senescence”) (Figure 4C; Supplementary Material 2). These results were in good agreement with the differences observed regarding changes in proliferative/mitotic activity between juvenile and adult retinas (see above). For example, the pathway related to “mitotic nuclear division” showed a high number of genes significantly changing their expression (Figure 4C).

Genes showing decreased expression in the adult retina (a positive fold change indicates decreased expression in adults) are good candidates to be pro-neurogenic and responsible for the higher proliferative activity of the juvenile retina. Actually, many of the genes that showed decreased expression in the adult retina [e.g., several cyclins (*Ccna2* or *Ccnb1*), *Ki67, N-myc, Otx2, Foxo1, Olig2, Ins1b, Mcm2, Meis2, Zic2, H2b2e, H3.2, Eomes* (*Tbr2*) or *Sox* family factors like *Sox8* or *Sox11* (Figures 4B,D; Supplementary Material 1)] are known to be important players in the maintenance of neural stem cell properties, cell proliferation, cell cycle progression or neurogenesis. Furthermore, genes belonging to signaling pathways known to play major roles in nervous system development (e.g., *Shh, Wnt, Notch* or *Slit-Robo* signaling pathways) also showed differential expression (Supplementary Table 1). These results were in good agreement with the histological and immunohistochemical observations (see above) and revealed that these signaling pathways might play a key role in retinal neurogenesis in sharks.

## Discussion

The teleost retina shows continual growth throughout life (Marcus et al., 1999). In sharks, the eyes also show continuous growth, with a close relationship between eye diameter and body length (Litherland et al., 2009; Collin, 2018). The organization of the peripheral retina observed in juveniles (present results; Ferreiro-Galve et al., 2010) was very similar to that previously described in late embryos (stages 33–34 as defined by Ballard et al., 1993; Ferreiro-Galve et al., 2010). However, in adults the peripheral retina showed a reduction in size compared to juveniles and also relative to the entire size of the adult retina. This decrease in the extension of the peripheral retina has been also observed during retinal development in *S. canicula* embryos (Ferreiro-Galve et al., 2010). The organization of mature cell types in different cell layers in the central retina in juveniles and adults was similar to that described in stage 33–34 embryos (Ferreiro-Galve et al., 2010). However, a decrease in the thickness of the INL, an increase in the thickness of the IPL and a decrease in cell density in the INL and GCL were observed in adults. This suggests that the increase in retinal size during postnatal development might not be mainly due to addition of new cells but rather to morphogenetic movements such as tissue narrowing by cell intercalation and extension and also to an increase in the extension/number of neuronal processes in the IPL. In goldfish (teleost), most of the adult retinal growth (~80%) is due to hypertrophy or expansion (Johns, 1977). In *Xenopus*, together with sustained cell production at the CMZ, a passive area expansion contributes to the overall retinal growth from the metamorphic climax to adulthood (Straznicky and Hiscock, 1984). Passive stretching has been also suggested to be involved in retinal growth in squamates (Eymann et al., 2019), birds (Fischer and Reh, 2000 and references therein), and mammals (Kuhrt et al., 2001).

Based on data obtained from teleosts (goldfish: Johns, 1977; rainbow trout: Julian et al., 1998; zebrafish: Marcus et al., 1999), it has been largely assumed that the retina of adult fishes presents cell proliferation and neurogenesis from CMZ progenitors and Müller glia (reviewed by Alunni and Bally-Cuif, 2016). Our data shows that the catshark retina has virtually no mitotic activity after sexual maturation, which together with histological data, suggests that retinal growth in sharks is caused by tissue expansion. The highly reduced TZ with almost no DCX positive neuroblasts in adults also indicates a lack of active neurogenesis. Lack of cell proliferation has been also observed in the adult lamprey retina (Villar-Cheda et al., 2008). Based on results on adult neurogenesis from the CMZ in teleost fish, amphibians, reptiles, birds, and mammals (see introduction) it has been suggested that the CMZ cells have been gradually diminished through the course of vertebrate evolution (Kubota et al., 2002). Our results in sharks suggest that a reduced neurogenic activity after sexual maturation could correspond to the ancestral character in jawed vertebrates.

In the peripheral retina (CMZ and TZ), we also observed significant changes in the expression of progenitor markers. The number of GFAP+ and *ScSox2*+ progenitors in the adult peripheral retina become highly reduced. Since the vast majority of peripheral cells do not express PCNA or pH3 at this period, they could correspond to quiescent progenitors that keep their ability to re-enter the cell cycle in case of injury or disease, as previously described for Müller cells in *Xenopus* (Langhe et al., 2017). Future work should determine whether mitotic activity, proliferation, and neurogenesis are reactivated after injury in adult catsharks.

Finally, RNA-Seq analyses of the retinal transcriptome of juvenile and adult catsharks revealed a decrease in the expression of genes typically associated with proliferation, neurogenesis, cell-cycle regulation, and maintenance of progenitor cell properties. This confirmed that the catshark retina losses most of its proliferative and neurogenic activity after sexual maturation and that these genes probably play key roles in maintaining a high neurogenic activity in juveniles. We also observed changes in the expression of genes belonging to pathways known to be involved in the control of neurogenic processes like *Shh, Wnt, Notch* or *Slit/Robo* signaling (Supplementary Table 1). The RNA-Seq results fit perfectly with the histological and immunofluorescence data. Taken together, these data and those reported in other vertebrates show that the maintenance of an active proliferating state in the adult retina is a variable trait of vertebrates, suggesting a high evolvability of underlying mechanisms. Our RNA-Seq data provide an excellent resource to identify new genes and signaling pathways controlling neurogenesis in the vertebrate retina, to decipher the molecular basis for such variations and clarify underlying evolutionary modalities.

## Data Availability Statement

The datasets presented in this study can be found in online repositories. The names of the repository/repositories and accession number(s) can be found at: https://www.ncbi.nlm.nih.gov/bioproject/688266, PRJNA668789.

## Ethics Statement

The animal study was reviewed and approved by Bioethics Committee of the University of Santiago de Compostela.

## Author Contributions

IH-N, FA, AB-I, and EC: conceived and devised the study, approved the article, and study concept and design. IH-N, SM, LS, HM, and DR: acquisition of data. IH-N, DR, FA, AB-I, and EC: analysis and interpretation of data and drafting of the manuscript. IH-N, SM, LS, HM, DR, FA, AB-I, and EC: critical revision of the manuscript. IH-N and EC: obtained funding. All authors had full access to all the data in the study and take responsibility for the integrity of the data and the accuracy of the data analysis.

## Conflict of Interest

The authors declare that the research was conducted in the absence of any commercial or financial relationships that could be construed as a potential conflict of interest.

## Supplementary Material

Supplementary Material 1 Differentially expressed genes between juvenile and adult retinas. A positive fold change indicates a decreased expression in the adult retina.

Supplementary Material 2 Gene Ontology (GO) and Kyoto Encyclopedia of Genes and Genomes (KEGG) pathway enrichment analyses. BP, biological process; CC, cellular component; MF, molecular function.

Supplementary Table 1 Genes belonging to developmental signalling pathways that showed differential expression in the RNA-Seq analyses.
